# Screening program for neonates at risk for developmental dysplasia of the hip: comparing first radiographic evaluation at five months with the standard twelve week ultrasound. A prospective cross-sectional cohort study

**DOI:** 10.1007/s00264-018-4089-2

**Published:** 2018-08-18

**Authors:** Dorien Geertsema, Joris E. Meinardi, Dagmar R. J. Kempink, Marta Fiocco, Michiel A. J. van de Sande

**Affiliations:** 1 Department of Orthopaedics, Leiden University Medical Center, Albinusdreef 2, Postzone J11, PO Box 9600, 2300 RC Leiden, The Netherlands; 2 Medical Statistics, Department of Biomedical Data Sciences, Leiden University Medical Center, Leiden, The Netherlands; 3 Institute of Mathematics, Leiden University, Leiden, The Netherlands

**Keywords:** Hip dysplasia, Treatment, Screening, Ultrasound

## Abstract

**Purpose:**

To assess whether delayed radiological hip screening at five months (versus ultrasound at 3 months) results in a higher incidence of persistent developmental dysplasia of the hip (DDH) at 18 months.

**Methods:**

We analyzed 3536 screened neonates (2009–2013) at age two to three weeks. In the case of risk factors for DDH, 460 infants were assigned to a pelvic radiograph at five months between 2009 and 2010 and 651 infants were assigned to an ultrasound at three months (2011–2013). In the case of DDH, appropriate treatment was started and radiological follow-up occurred at eight, ten, 12, and 18 months. We compared incidence and severity of persistent DDH at 18 months. Analysis was performed using linear regression.

**Results:**

Both groups were comparable for risk factors (breech, gender, twins, family history). Eighty-nine patients (2.5%) showed DDH (*n* = 43 (group 1), *n* = 46 (group 2)). At 18 months, ten patients showed persistent DDH (*n* = 8 (group 1), *n* = 2 (group 2) (7.7% vs. 0.3% respectively)). The mean acetabular index (AI) at 18 months in group 1 (left hip) is 22.4° (95% CI 20.6–24.3°) vs. group 2 at 22.3° (95% CI 21.2–23.4°) (*p* = 0.098). The mean AI in group 1 (right hip) is 21.9° (95% CI 18.9–24.9°) vs. 21.2° (95% CI 20.5–22.0°) in group 2 (*p* = 0.293). Adjusted for risk factors, there is no difference in incidence of persistent DDH between both groups after 18 months (OR 0.519; 0.07, 3.845).

**Discussion:**

This study revealed no significant difference in incidence or severity of persistent DDH at 18 months between the two screening groups. These results suggest justification for delayed screening to prevent overtreatment of immature hips.

**Conclusion:**

In clinically stable hips, delayed ultrasound between three and five months is regarded as safe and could prevent for overtreatment of mild dysplastic hips.

## Introduction

Developmental dysplasia of the hip (DDH) is a relatively common condition with an incidence of around 10/1000 (1%) in the newborn population. Bialik et al. even reported an incidence of 55.1/1000 after hip ultrasound (US) screening according to Graf [1], but only 5/1000 required treatment, which represented the true incidence. The study also mentioned the wide variety and discrepancy in defining DDH in literature due to different diagnostic criteria. Dislocation of the hip is relatively rare with an incidence of 1 to 2 per 1000 (0.1%).

Although ultrasound (US) is most commonly used to screen neonates for DDH, discussion remains on the optimal screening methods to diagnose DDH. Timing is one of the most striking differences in the screening programs implemented around the world, where some countries screen at the age of two weeks and others prefer screening at later ages [2–4] (https://www.help.gv.at/Portal.Node/hlpd/public/content/8/Seite.082200.html). In addition, a clear definition of an “abnormal” hip US is lacking. Bialik et al. showed that 90.4% of the hips that were sonographically positive for DDH at birth normalized without receiving treatment at an age of one year [1].

Therefore, we set out to evaluate the optimal timing of radiological screening in order to optimize the sensitivity of the screening program and minimize subsequent over- or under-treatment.

The Dutch national screening program is well established and implemented since the beginning of the 1980s. All newborns are clinically screened within the first two weeks after birth and thereafter at fixed timeslots (*consultation bureau for neonates*). In the case of abnormalities during physical examination, and/or if any absolute risk factors (breech position during pregnancy, gender, twin birth, and a positive family history of DDH) are present, the newborn is subjected to a hip ultrasound (US) examination and a consultation by a paediatric orthopaedic surgeon as described by the Dutch guidelines [2, 3].

As a result, not every newborn receives standard hip US, whereas in many other European countries (Germany, Austria, and Switzerland), this is standard of care. In Germany, a standard hip US is performed at an age of four to five weeks (so called U3). In Austria and Switzerland, the screening is performed in the first week of life, but in Austria, the hip US is then repeated in week six, seven or eight [4, 5] (https://www.help.gv.at/Portal.Node/hlpd/public/content/8/Seite.082200.html). The American Academy of Pediatrics (AAP) advises that every newborn is to be screened by physical examination and that the screening is done by a properly trained health care provider (e.g., physician, paediatric nurse practitioner, physician assistant, or physical therapist). US of all newborns however is not recommended. Only if physical examination is positive (e.g., Ortolani/Barlow) at two weeks of age is referral to an orthopaedic center advocated for physical examination with or without US depending on present risk factors. A standard pelvic radiograph is not recommended because they are of limited value, give a low dose of radiation, and do not influence treatment decisions at this age. Although relatively low in cost, only after four to six months when the ossification center of the femoral head becomes visible could this be of more diagnostic value in the evaluation and therapy of DDH [6]. These differences again underline the need for an evaluation of optimal timing of radiographic screening for DDH in infants.

Our orthopaedic department provides a weekly open consultation-screening program where newborns are screened at an age of two to three weeks and undergo a clinical evaluation and assessment of possible risk factors performed by a paediatric orthopaedic surgeon or orthopaedic resident. If required, an US is performed at six or 12 weeks depending on the findings at physical examination (i.e., instability) [2].

Over the last ten years, a large prospective database was built up in which we collected clinical and radiological data to get a better insight in the screening methods, treatment choices, and incidence of persistent DDH at a later age. According to the current literature and guidelines, we switched from radiographic to ultrasound screening in 2011. The aim of this study is to assess whether an expedited radiological hip screening with ultrasound at three months (versus a radiograph at 5 months) results in a lower incidence of persistent DDH at 18 months. We therefore prospectively evaluated two different screening protocols, radiographic evaluation at five months versus ultrasound evaluation at three months.

## Materials and methods

Between January 2009 and December 2013, we screened a total of 3536 neonates (7072 hips) at an age of two to three weeks. Two thousand three hundred and thirty-three (66%) neonates were discharged after screening according to the national screening guidelines (none of the aforementioned absolute risk factors and normal physical examination). Ninety-two neonates (3%) showed clinical abnormalities (e.g., leg length discrepancy, + Barlow, + Ortolani) and were allocated to an US at six weeks of age. This group was out of scope for this study and subsequently excluded from this study.

Between 2009 and 2011, 460 neonates (920 hips) with risk factors (not instability) for DDH were assigned to a pelvic radiograph at five months (group one). For each follow-up interval, the acetabular index (AI) was measured and radiographic evaluation of the hips according to Tönnis [7] was performed and the presence of DDH was scored.

Between 2011 and 2013, 651 neonates (1302 hips) with known risk factors were assigned to a hip ultrasound (US) at three months (group 2). Alpha and beta angles were measured and the hips were defined according to the Graf classification system per hip [8, 9]. The diagnosis of DDH (≥ Graf 2b) was assigned to each hip separately based on these findings (left or right hip, or both hips: DDH yes/no).

Relative risk factors included presence of clubfeet, prematurity, birth weight, and oligohydramnios. These were regarded no reason for standard US hip screening after physical screening at two to three weeks.

All data was anonymously collected using the Project Manager Internet Server (ProMISe) database, which was especially designed for this purpose in cooperation with the Advanced Data Management Department at Leiden University. In this database, we prospectively collected all clinical and radiological data obtained during the screening at our outpatient clinic at first visit (age 2–3 weeks). If further screening was indicated, all clinical evaluations thereafter were documented until discharge.

In the case of DDH, which was determined either by radiograph or US, patients were treated with a hip abduction brace (a Camp or Pavlik harness).

After initial screening at five months of age, group 1 had a further radiographic follow-up at eight, ten, 12, and 18 months with registration of the AI according to Tönnis [7].

In group 2, the Graf type 2a (alpha angle 50–60; age ≤ 3 months) and type 2b (alpha angle 50–60°; age > 3 months) underwent repeated US after six weeks. In the case of Graf type 2c (alpha angle 43–49°; any age) or worse at any age (3 months or older), treatment was initiated with a Pavlik harness. During weekly visits, the Pavlik harness was checked and adjusted if necessary at the outpatient clinic. US evaluation was repeated after six weeks. If (sub)luxation persisted, closed reduction and spica casting were initiated. Standard radiographic follow-up and AI registration was performed at eight, ten, 12, and 18 month follow-up as well.

As principal outcome measure, we compared the presence and severity of persistent DDH at 18 months according to the Tönnis classification. To study the association between DDH and risk factors, a multivariate logistic regression model (the Wald test) with risk factors gender, breech position, twin birth, and positive family history was estimated. All statistical analysis was performed using SPSS version 20.0. Differences were considered significant if *p* < 0.005.

## Results

**Fig. 1:**
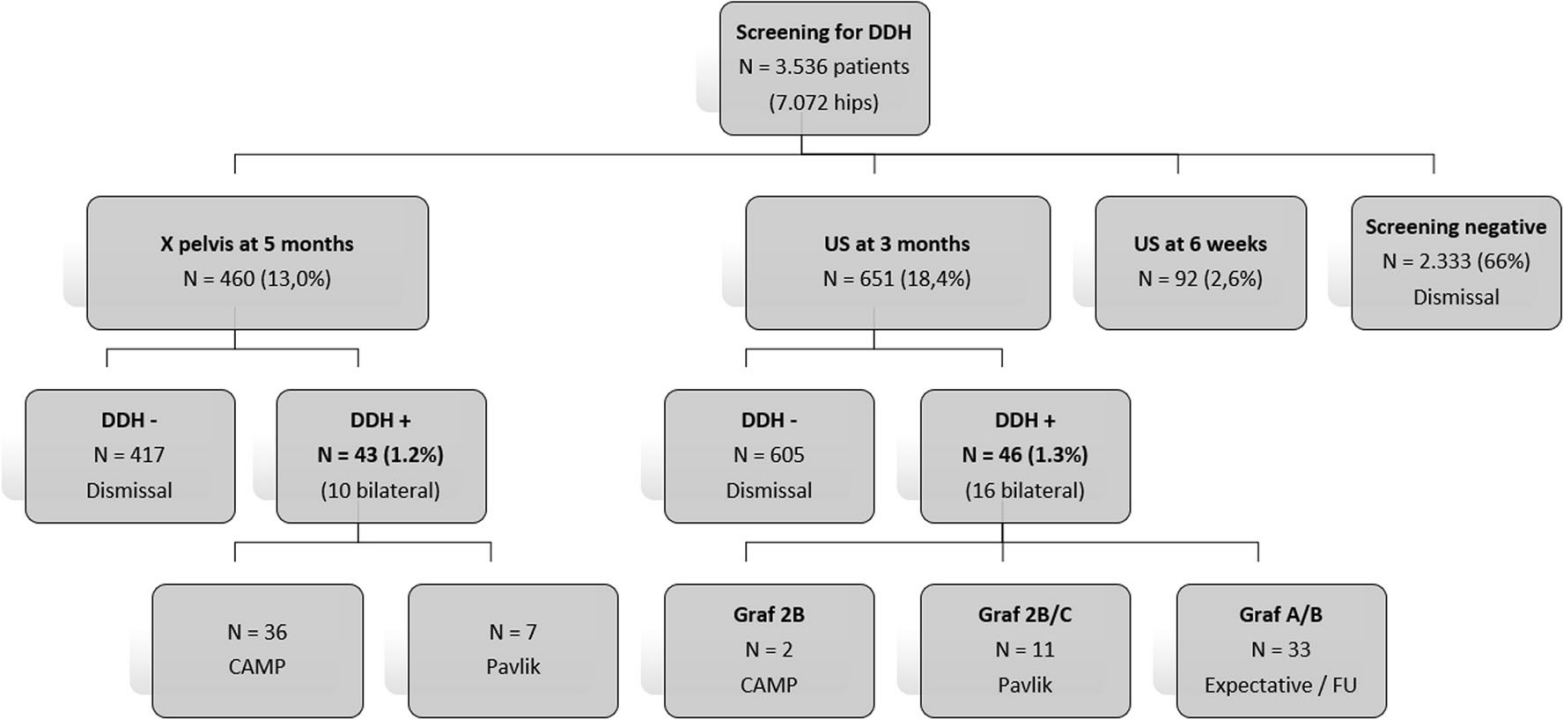
Treatment after screening for both groups. US: Ultrasound

Both groups were found comparable for absolute risk factors such as gender, breech position, twin birth, and positive family history. In group 1, 90.7% (417/460) of the children were discharged with normal radiographs. In group 2, 92.9% (605/651) had normal Graf 1 hips on ultrasound at three months. In group 1 (at 5 months), 43/460 children (53 hips) showed DDH of which 10/43 were bilateral. For the left hip, the mean AI was 33.4° (31–40°) and for the right hip, the mean AI was 33.2° (31–39°). In group 2 (at 3 months), 46/651 children (62 hips) showed DDH (bilateral 16). In Graf type 2b (*n* = 33), a “supervised neglect” policy was started. Thirteen children showed Graf 2c hips in which standard treatment was initiated with a Pavlik harness. The incidence of DDH in both groups is around 1%: in group 1 1.2% (43/3536) and 1.3% in group two (46/3536) (Table 1 and Fig. 1).

**Table 1:** No. of patients with rest DDH at screening and 18 months (*N*)

Screening group	DDH+ at screening	DDH+ at 18 months	Odds ratio
X pelvis at 5 months (*N* = 460)	43 (53 hips) (1.2%)	8 (8 hips) (1.7%)	0.519* (0.07–3.845) *p* = 0.565
US at 3 months (*N* = 650)	46 (62 hips) (1.3%)	2 (3 hips) (0.3%)	

*Corrected for gender, breech position, and positive family history

At 18-month follow-up, 8/460 children (7.7%) from group 1 showed mild persistent DDH (7 left, 1 right) (Table 1). One patient received a Camp treatment and regular follow-up. In group 2, 2/651 (0.3%) (3 hips: 2 right, 1 left) were still considered dysplastic (no dislocations) and were followed without bracing (see Fig. 1). The AI values for the two right hips were 25° and 27° and for the left hip 31°.

The mean AI at 18 months for the left hip did not differ between both groups: group 1, 22.4° (95% CI 20.6–24.3°) vs. 22.3° (95% CI 21.2–23.4°) in group two (*p* = 0.098). This was the same for the right hip: group 1, 21.9° (95% CI 18.9–24.9°) vs. 21.2° (95% CI 20.5–22.0°) in group two (*p* = 0.293; Table 2).

**Table 2:** Mean acetabular index (AI in degrees) at 18 months

Hip	X pelvis at 5 months	US at 3 months
Left	22.4 (95% CI: 20.6–24.3)	22.3 (95% CI: 21.2–23.4)
Right	21.9 (95% CI: 18.9–24.9)	21.2 (95% CI: 20.5–22.0)

At 18 months, we found no significant difference in incidence or severity of persistent DDH between both groups. In total, ten children showed mild remaining dysplasia according to Tönnis: eight (1.7%) from group one and two (0.3%) from group two. The odds ratio (OR) adjusted for gender, breech, family history was 0.519 (0.07–3.845, *p* = 0.565; Table 1). For all but one case, which was treated in a Camp abduction brace, regular follow-up occurred without bracing or surgical intervention. There was no significant difference in treatment duration comparing both screening groups (Table 3).

**Table 3:** Duration of treatment for DDH (months)

Screening group	Pavlik/CAMP^^^	CAMP	Total	95% CI*
X pelvis at 5 months	2.9	2.8	5.7	2.2–3.4
US at 3 months	2.4	2.5	4.9	1.8–3.1

*CI confidence interval, ^*CAMP* hip abduction brace

Although no dislocations were observed in either groups, three children were re-referred by the family doctor due to complaints. It appeared they all had a dislocated hip. They were further treated according to protocol and not included in the study results. Retrospectively, at initial screening at two weeks of age, there were no clinical signs or known risk factors for DDH or dislocated hips in these children.

## Discussion

This study evaluated two different screening protocols for diagnostics and treatment of DDH in one pediatric hospital in the Netherlands. We have found no significant difference in the number or severity of persistent DDH at 18 months between ultrasound evaluation at three months and radiographic assessment at five months. Therefore, one could argue that, in the case of positive risk factors at initial screening at two weeks, it is safe to follow up with ultrasound between the ages of three and five months. This policy shows no adverse effects in the short term where (sub)luxations were not found more often in one or the other group. The duration of treatment in a Pavlik harness or Camp abduction brace was also comparable. In addition to this, we found that the Graf 2b hips autocorrected over time which prevents overtreatment. This is supported by Bialik et al. [1]. Clinically abnormal hips at screening were left out of the scope of our study. We cannot emphasize enough that these hips need early sonographic screening in order not to miss hip dislocations and warrant direct treatment. It is essential that during US (according to Graf’s criteria), the examiner is aware of the risk of under- or overestimating the measured α-angle due to transducer inclination variations [10].

Considering the risk factors in the screened children in the entire cohort, we noticed that breech position and a positive family history dominated in group two (US group): 30% vs. 18% and 66% vs. 36% respectively. Gender was more or less equally distributed: girls 59% vs. 51% in group one and two respectively. In other words, the reason for referral was found more diverse in group one (pelvic radiograph). This can be explained by an updated guideline which contains clear criteria for referral to radiological examination [2].

Our results are in line with the outcomes presented by Rosendahl et al. They show the same trend even in Graf 2c hips: stable, so called mildly dysplastic hips did not show worsening in DDH and autocorrected over time with active surveillance. The only difference is that they had already screened the infants at two to three days. In this randomized controlled trial, they allocated 128 infants with Graf 2c (alpha angle 43–49°) at two to three days of age to either direct treatment or follow-up with a control US at six weeks [12]. This active surveillance halved the number of children requiring treatment, did not increase the duration of treatment, and yielded similar results at one and six year follow-up [11, 12]. Also, previous studies showed that immature hips undergo physiological auto-correction in the first two to three months of life in which the alpha angle (acetabular inclination) reaches a plateau [2, 13]. Thus, treatment probably accelerates this physiological auto-correction but active surveillance does not lead to a higher incidence of DDH at one year of age [2, 13].

In the Netherlands, we clinically screen infants at an age of two to three weeks and then perform an US at six weeks in case of clinical instability or at 12 weeks when the infant carries risk factors. Our results underline the reassuring hypotheses that performing an US (or a radiograph) at even a later age than six weeks (12 weeks or 5 months) can result in similar results for the incidence and severity of persistent DDH at 18 months. Thus, the 12-week US in infants with stable hips at first clinical examination could be postponed with one or two months as it will not only have implications for therapy, health care costs, discomfort of infants, and parents, but also reduces the risk of complications (e.g., osteonecrosis of the femoral head, femoral nerve palsy).

If one postpones screening to an age of five months in infants without risk factors and stable hips at clinical screening, the question arises as to whether the radiological evaluation should include a radiograph or ultrasound. Femoral head calcification starts at around two to eight months of age so it could be potentially more difficult to evaluate the deepest part of the acetabulum due to scattering but clearly there is a wide variation.

The American College of Radiology states that US is the preferred modality for evaluating the hips in infants who are six months or younger [14]. This would imply that a plain pelvic radiograph would not be needed, preventing exposure to radiation. If US is unsuccessful, then a plain pelvic radiograph could follow. Vedantam et al. published a protocol for only selective US with a screening radiograph at five months for any child cleared by US at six weeks. They found no abnormalities from these radiographs [15]. In the follow-up after closed reduction, for instance, Li et al. described that the AI is the best predictor of late residual acetabular dysplasia [16].

Is US a good alternative or even a preferred method to evaluate hips at four to five months and does it provide the same information about the acetabular coverage compared to a radiograph? To our knowledge, no study ever investigated this question and little is known about “late” US. Roovers et al. though showed that “late” US (at 8 months) did not lead to a significant difference in the proportion of cases that were detected late with clinical screening compared with ultrasound screening [17]. Malkawi et al. showed that an initial US at four months might prevent the overtreatment of auto-correctable hips detected with early US (within 12 hours after birth) [18]. In this study, it was unclear whether clinical screening was done before the US. Additionally, Pillai et al. showed that the accuracy of US is lowest before six weeks of age and highest at three months. The specificity showed the same trend (56% at 6 weeks and 96% at 3 months). This study showed that there was no significant difference between US (at 0–4 weeks, 6 weeks, and 3 months) and pelvic radiograph at six months of the same hips looking at accuracy, sensitivity, specificity, positive predictive value, negative predictive value, and the positive and negative diagnostic likelihood ratio [19]. We also saw this in our groups: US and pelvic radiograph accurately predict the *severity* of DDH.

Clearly, clinical screening at two to three weeks should be performed by an experienced physician able to detect clinical abnormalities. If there is any doubt, an US should be performed at six weeks to detect a dislocated/dislocatable hip. In the absence of any sign of instability but with known risk factors and looking at our results, we would opt that it is safe to perform an US at four to five months to detect the “true” cases of DDH and prevent overtreatment of immature stable Graf 2a hips. In this way, Graf 2b/c hips are not overlooked and treated accordingly. In our experience, US can reliably be implemented as the screening method until four to five months. This may well be followed by radiological evaluation in selected cases. In their systematic review, Woolacott et al. opted for good quality trials in which timing of US at one and three months as well as the treatment and management should be incorporated [20]. We would opt to study the addition of US at four and five months versus pelvic radiographs at the same time. Only then can the non-inferiority, safety, and value of US at four to five months be tested reliably and show whether pelvic radiograph is still of value in the diagnostics and early follow-up in DDH. Postponed screening at an age of four to five months would implicate that any necessary treatment is initiated at an older age. Treatment duration might be extended due to the decreasing capacity of the acetabulum to remodel over time, although we have not seen this trend in our study. Even then, in DDH cases at a later walking age (12–18 months), Cha et al. showed that the results of stable and concentric closed reduction may provide similar results compared to the non-walking children if performed with preliminary gentle traction (to decrease the risk of AVN), percutaneous adductor tenotomy, and minimization of forceful abduction. The impact of traction for favourable outcomes in this study is debatable [21]. Yong et al. also addresses that a MRI is mandatory after closed reduction and spica casting in the age of six to 18 months to prevent missing posterior luxations on a standard pelvic radiograph, regardless of subspecialty, level of expertise, and geographical origin [22].

To conclude, this large prospective cohort study revealed no significant difference in incidence rates or severity of persistent DDH at 18 months between two follow-up protocol groups (radiograph at 5 months vs. US at 3 months). These results suggest a justification for delayed ultrasound screening (age 4–5 months) to prevent overtreatment of immature hips. Paramount is clinical examination in experienced hands at two to three weeks of age to detect unstable or dislocated hips that need to be examined with an US at six weeks. In clinically stable hips but with present risk factors, an US at four to five months is regarded as safe and can prevent overtreatment of mild dysplastic hips.
